# Peptide charge state determination of tandem mass spectra from low-resolution collision induced dissociation

**DOI:** 10.1186/1477-5956-9-S1-S3

**Published:** 2011-10-14

**Authors:** Jinhong Shi, Fang-Xiang Wu

**Affiliations:** 1 Division of Biomedical Engineering, University of Saskatchewan, Saskatoon, S7N 5A9, Canada; 2 Department of Mechanical Engineering, University of Saskatchewan, Saskatoon, S7N 5A9, Canada

## Abstract

**Abstract:**

**Background**

Charge states of tandem mass spectra from low-resolution collision induced dissociation can not be determined by mass spectrometry. As a result, such spectra with multiple charges are usually searched multiple times by assuming each possible charge state. Not only does this strategy increase the overall database search time, but also yields more false positives. Hence, it is advantageous to determine charge states of such spectra before database search.

**Results:**

We propose a new approach capable of determining the charge states of low-resolution tandem mass spectra. Four novel and discriminant features are introduced to describe tandem mass spectra and used in Gaussian mixture model to distinguish doubly and triply charged peptides. By testing on three independent datasets with known validity, the results have shown that this method can assign charge states to low-resolution tandem mass spectra more accurately than existing methods.

**Conclusions:**

The proposed method can be used to improve the speed and reliability of peptide identification.

## Background

Mass spectrometry has been widely used to analyze high throughput protein samples. Proteins are first cleaved into peptides with enzymes or chemical cleavages. Then, peptides are separated from mixture solutions by high pressure liquid chromatography (HPLC), and sent to ionization sources where they get ionized. There are two ionization techniques, electrospray ionization (ESI) and matrix assisted laser desorption/ionization (MALDI), which are often used in proteomics laboratories. MALDI is mainly used in peptide mass fingerprinting as it predominantly yields singly charged ions. Unlike MALDI, ESI typically produces multiply charged ions. After being ionized, peptides are introduced into analyzers such as ion trap or triple quadrupole to produce mass spectra (MS). To obtain tandem mass spectra (MS/MS), peptide ions with the highest intensities in MS are isolated and subjected to fragmentation by collision induced dissociation (CID). The resultant MS/MS are used to provide structural composition information of peptides.

The commonly used database search programs for peptide identification include Sequest [1] and Mascot [2]. These programs compare experimental spectra with theoretical spectra in a database and use scoring functions to measure the similarity between them. Typically, the peptide with the highest score is identified. However, the growing number of protein sequences in expanding databases becomes a challenge for database search software because the search space is sharply increasing. Moreover, multiply charged peptide tandem mass spectra from ESI-CID also add complexities to these programs, because they generate much more complex spectra. Although high-resolution mass spectrometers can provide separable isotropic spacing of fragment ions to derive charge states, most commonly used ion trap and triple quadrupole analyzers have limited resolution to do so [3]. In such a case, one spectrum is usually searched multiple times by assuming each possible charge state of its precursor peptide ion. This strategy increases the overall time of database search and yields more false positives as true positives need to be distinguished from much more peptide candidates. The requirement of determining peptide charge states is not limited to database search, but also is necessary in de novo sequencing methods [4].

This paper will focus on the charge state determination of low-resolution tandem mass spectra. There have been reports in determining charge states of low-resolution tandem mass spectra [3,5-7]. Thirty-four features were proposed in [5] to describe MS/MS and the link between MS and MS/MS, then support vector machine (SVM) was used to classify MS/MS into three groups +2, +3 and +2/ +3. One problem with this method is that it classifies peptide ions into three groups, which still leaves ambiguities in the charge determination. Lately, twenty-eight features of MS/MS were proposed to train SVM in [7] to discriminate doubly and triply charged peptides. The common problem with [5,7] is that SVM needs trained with labeled data. This inherent drawback of supervised methods limits their generality in determining the charges of any experimental MS/MS. Last but not least, it is computationally expensive to first train SVM and then apply it on test data.

In this paper, we present an unsupervised learning method based on Gaussian mixture model (GMM) to determine the charge states of low-resolution tandem mass spectra. Four novel and discriminant features are proposed to describe MS/MS. By testing on three low-resolution MS/MS datasets with verified charge states, the results have shown that the proposed method can accurately assign charge states to such tandem mass spectra.

## Methods

In database search, tandem mass spectra are usually considered to carry 1, 2 and 3 charges. Research [8] shows that singly charged MS/MS can be reliably determined. Therefore, the charge state determination can be reduced to the classification of doubly and triply charged MS/MS. To solve this problem, this study uses the unsupervised GMM with features proposed to reflect the properties of MS/MS. Since the features are to be extracted from MS/MS, we will first introduce several properties of peptide CID tandem mass spectra. For more details about these properties, we would refer readers to [9].

### Properties of CID tandem mass spectra

Let *m*(a*_i_*) be the mass of amino acid a*_i_*, then the mass of peptide P with *n* amino acids is given by(1)

where *m*(H) and *m*(OH) are the masses of the additional N-terminal and C-terminal. The cleavage along peptide bonds in CID mainly leads to the production of N-terminal b*_i_* ion and C-terminal y*_n_*_–_*_i_* ion. The singly charged ion with N-terminal is denoted by , and its *m*/*z* value is(2)

The *m*/*z* value of its doubly charged counterpart  is(3)

The singly charged ion with C-terminal is denoted by , and its *m*/*z* value is(4)

Here two hydrogens are added because C-terminal ion carry one negative charge after fragmentation, thus it needs two protons to make it carry one positive charge. Similarly, the *m*/*z* value of its doubly charged counterpart  is(5)

From equations (1) to (5), we have the following equations holding for peptide CID tandem mass spectra:(6)(7)(8)(9)

Since one peptide with different charges can produce different MS/MS, we can infer the charge state of a peptide according to the features of its MS/MS. As we will see, these features will be calculated based on the above relationships between the singly and doubly charged fragment ions.

### Spectrum features

First, six variables are defined for a given peptide MS/MS [9] as follows:

where *m*_1_ and *m*_2_ are the *m*/*z* values of any two peaks from the given peptide tandem mass spectrum and *m*_2_ >*m*_1_.

#### Complementary pairs

Complementary pairs measure the likelihood that an N-terminal ion and a C-terminal ion in a peptide MS/MS are produced as the peptide fragments at the same peptide bond. Let

then, the first feature is defined as(10)

where |·| denotes the cardinality of a set. The feature *δ*_cp_ is the difference between the number of complementary pairs (+1, +1) and the number of complementary pairs (+1, +2) in MS/MS. This feature accounts for the fact that +2 peptides tend to generate two +1 ions at the same bond, while +3 peptides are prone to yield one +1 and one +2 ion [3,6]. From the definition, this feature is expected to be larger for doubly charged peptides than triply charged ones.

According to the definition of *s*_1_, *s*_2_ and *s*_3_, we define peak sets

Then, the second feature is given by(11)

where *I*(·) represents the intensity of peaks. The feature *δ*_R_cp__ is the difference between the ratio of +1 peak intensity over their complementary +1 peak intensity and the ratio of +2 peak intensity over their complementary +1 peak intensity. The item 0*.*5 is added in view that the intensity of *y* ions in higher mass regions is larger than that of *b* ions in lower mass regions. This feature accounts for the fact that the intensity of +1 peaks and the intensity of their complementary +1 peaks should be comparable when they are produced from doubly charged peptides, while the intensity of +1 peaks from triply charged peptides should be comparable to the intensity of their complementary +2 peaks. Thus, the difference between these two ratios should be greater than 0 for doubly charged peptides while less than 0 for triply charged ones. This newly proposed feature is expected to be more significant than the first feature proposed in [3], because it integrates the intensity information into the feature definition rather than just counts the number of complementary pairs.

#### Regional intensity

Intensity is an important property of tandem mass spectra, so we incorporate it into the expression of the third feature. Let

then according to the definition of *d*_1_, *d*_2_, *d*_3_, we can see that the set of doubly charged peaks is

In view of further manipulation, we define an indicator function of the peak masses in a spectrum,

where *m_p_* is the *m*/*z* value of parent peptide ions. Then the third feature is defined as(12)

The feature *I*_dc_ is the intensity of +2 peaks in the mass region [*m_p_*, 1*.*5*m_p_*]. In theory, the *m*/*z* values of +2 peaks from +2 peptides should not exceed *m_p_*, while they should not exceed 1*.*5*m_p_* when they are from +3 peptides. Hence, *I*_dc_ which accounts for the +2 peak intensity in the region [*m_p_*, 1*.*5*m_p_*] should be very discriminant for doubly and triply charged peptides. This feature is expected to be smaller for doubly charged peptides than triply charged ones.

#### Amino acid distance

The charge state of a peptide is theoretically determined by the number of basic amino acids it contains [10]. The side chains of basic sites have high proton affinities to attract protons in ESI, and the N-terminal amine group can also attract a proton. Thus in theory, doubly charged peptides should contain one basic site and triply charged peptides should contain two basic sites. Let *n*_bs_ be the number of basic sites of an MS/MS, and define

then the number of basic sites is computed by(13)

where *N_t_* is the theoretical repeat number of basic residues in a mass spectrum. More discussion about *n*_bs_ is given later.

When we compute the values of all features, the situations when peaks are produced by losing water, ammonia, CO or NH group are considered as proposed in [7].

### Gaussian mixture model

Gaussian mixture model (GMM) is commonly used for clustering and it is unsupervised, which makes GMM have an obvious advantage over other supervised methods in terms of saving efforts in labeling training data. The expression of Gaussian mixtures is given by(14)

where(15)

and *p_k_* is the mixing probability of the *k*^th^ component. Here, *D* is the space dimension of data points. The maximum likelihood approach is used to estimate the parameter vector *θ* in GMM. The likelihood function is given by(16)

Substituting the Gaussian mixtures (14) into (16), and taking the logarithm of the likelihood function, we have(17)

Then, the parameter *θ* is given by(18)

To solve (18), we take the derivatives of *L* with respect to *µ_k_* and *σ_k_*, which yields(19)(20)

where(21)

In the above expression, *p*(*k*, *n*) is defined as(22)

Note that the volume *d***x** cancels in (21). To obtain the derivative of *L* with respect to the mixing probability *p_k_*, we write the variables *p_k_* as functions of unconstrained variables *γ_k_*[11], given in (23), because the values of *p_k_* are constrained to being positive and adding up one.(23)

This transform enforces both constraints automatically. From the chain rule of differentiation, we obtain(24)

Setting all derivatives to zero, we obtain three groups of equations for the means, variances, and mixing probabilities:(25)(26)(27)

These equations are intimately coupled with one another, because the term *p*(*k|n*) in turn depends on all terms on the left-hand sides through (21) and (22). Thus, it is hard to solve these equations directly. However, EM algorithm can provide a solution. We start with a guess for the parameters *p_k_*, *µ_k_*, *σ_k_*, and then iteratively cycle through (21), (22) (E-step), and then (25), (26) and (27) (M-step). The procedures of EM algorithm are given as follows:

• E-step:(28)

• M-step:(29)(30)(31)

## Results and discussion

### Experimental data

Three datasets are used to investigate the performance of the proposed method in predicting charge states of peptide CID tandem mass spectra.

• ISB dataset ISB dataset was acquired on an LC-ESI ion trap (ThermoFinnigan) and was provided by the Institute of Systems Biology (ISB, Seattle, USA). It contains 37,044 peptide MS/MS from a control mixture of 18 standard proteins [12]. The charge states were assigned to 1656 doubly charged and 984 triply charged peptides with Sequest.

• TOV dataset TOV dataset includes 22,577 peptide MS/MS which were acquired on an LCQ DECA XP ion trap (Thermo Electron Corp.). The samples analyzed were generated by the tryptic digestion of a whole-cell lysate from 36 fractions of TOV-112D [13]. These spectra were searched using Sequest and the assignments of 1898 doubly charged and 261 triply charged spectra were verified to be correct by Scaffold (http://www.proteomesoftware.com) with the minimum probability of 0*.*95.

• BALF dataset BALF dataset was obtained from an LCQ DECA ion trap mass spectrometer (ThermoFinnigan) and is available in PeptideAtlas (http://www.peptideatlas.org/repository) data repository. MS/MS were searched with Sequest against IPI human protein database. The assignments of 2492 doubly charged and 3686 triply charged spectra were validated using PeptideProphet with the minimum probability 0*.*90.

### Results

GMM is solved by implementing the EM algorithm described previously with MATLAB. All features are transformed to have variances 1. Receiver Operating Characteristic (ROC) curve and Area Under the Curve (AUC) are employed to measure the classifier performance. ROC curves of actual classifications locate in between the ideal plot (the point (0,1)) and the random-guess plot (the diagonal line) with AUC ∈ (0*.*5, 1). The bigger the AUC, the more powerful the classification is.

#### Comprehensive performance of the features

**Figure 1:**
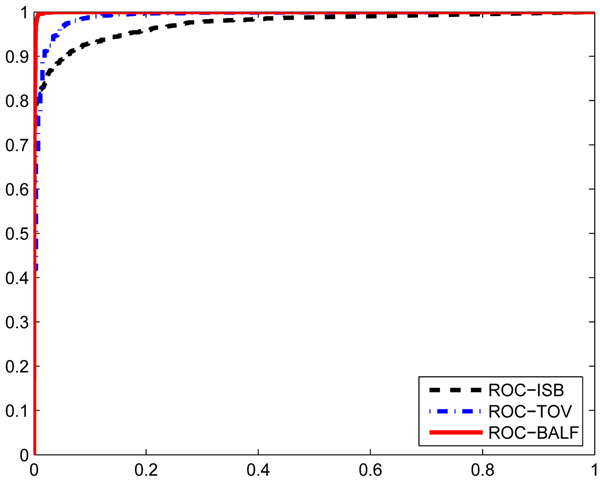
**ROC curves with all features.** ROC curves of ISB, TOV, and BALF data with all features. AUC_ISB_ = 0*.*9732, AUC_TOV_ = 0*.*9903, AUC_BALF_ = 0*.*9990.

First, we build the classifier with all features to see their comprehensive performance. The estimated means of the four features for doubly and triply charged peptides of the three datasets are shown in Table 1. It can be seen that all these estimated values are consistent to the expected values. ROC curves of the three datasets are given in Fig. 1. AUC for ISB, TOV and BALF are 0*.*9732, 0*.*9903, 0*.*9990, respectively. Both ROC and AUC show that GMM with the proposed features is well-suited for the classification of low-resolution peptide CID tandem mass spectra.

**Table 1:** Estimates of means of all features and their expected relationships

Features	ISB		TOV		BALF		EXPECTED Feature values
	+2	+3	+2	+3	+2	+3	
*δ*_cp_	–0.0956	–1.5366	–0.4592	–2.1642	–0.8590	–2.3805	+2 > +3
*δ*_Rcp_	0.8384	–0.5340	0.8842	–0.4470	0.4762	–1.3666	+2 > +3
*I*_dc_	0.2099	1.4521	0.3941	2.0239	0.4743	1.5057	+2 < +3
*n*_bs_	0.4887	1.4556	0.9962	2.1185	1.2003	1.2302	+2 < +3

Estimates of means of all features for +2 and +3 MS/MS and their expected relationships.

#### Discriminant power of each feature

Here we examine the power of each proposed feature in discriminating doubly charged and triply charged peptides with AUC, which is given in Table 2. The AUC shows that the most significant feature is *δ*_R_cp__, which measures the comparable degree of the intensity of complementary pairs. The second one is the commonly used feature *δ*_cp_ and the third one is *I*_dc_, which accounts for the intensity difference of doubly charged peaks in the mass region [*m_p_*, 1*.*5*m_p_*]. The feature with the least discriminant power is the number of basic sites *n*_bs_. Theoretically, this feature reflects the origin of the charges carried by peptides through ESI, thus it should be significant in distinguishing doubly and triply charged peptides. More discussions are given for this inconsistent result in the following subsection.

**Table 2:** AUC of classifiers built with each feature

	ISB	TOV	BALF
*δ*_cp_	0.9832	0.9839	0.9613
*δ*_Rcp_	0.9905	0.9856	0.9964
*I*_dc_	0.8973	0.9268	0.8190
*n*_bs_	0.6624	0.6476	0.5124

AUC of classifiers built with each feature for the three datasets.

**Figure 2:**
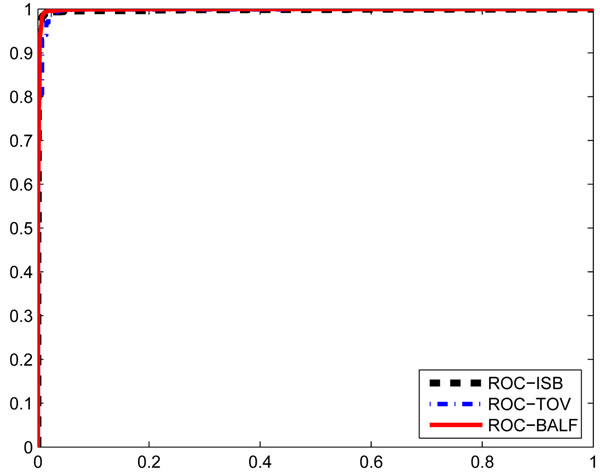
**ROC curves with three most significant features**. ROC of ISB, TOV, and BALF with three most significant features. AUC_ISB_ = 0*.*9976, AUC_TOV_ = 0*.*9970, AUC_BALF_ = 0*.*9984.

The three most significant features are used to build the GMM classifier and the performance is given in Fig. 2. It is obvious that the classifier is very powerful in separating doubly charged and triply charged peptides in all three datasets. Furthermore, it is even better than the classifier built with all features.

#### Comparison with existing methods

Since the number of basic sites is not finally determined, we compare the results given in [6] with our results obtained with the other three features, which is shown in Table 3. By testing on the same ISB dataset, the proposed features can achieve both higher precisions for doubly and triply charged MS/MS as well as a higher accuracy for all spectra. This indicates that the three features are significant in discriminating doubly charged MS/MS from triply charged ones. Besides, testing these features on the other two independent datasets indeed verify their discriminant power.

**Table 3:** Caparison with the results given in [6] on the same ISB dataset

Features		Estimated Parameters		Precision		Accuracy
			
		+2	+3	+2	+3	
	*δ*_cp_	–0.1175	–1.8433			
				
GMM	δ_Rcp_	0.8228	–0.8352	0.9803	0.9886	0.9833
				
	*I*_dc_	0.2847	1.6196			

SVM	see [6]	N/A		0.9240	0.9380	0.9310

Results obtained by using three features on ISB dataset and the caparison with the results given in [6] on the same dataset are provided.

#### Discussion of the number of basic sites

The result about the discriminant power of each feature shows that the number of basic sites is not powerful in discriminating peptides with different charges. The reason is that the computation of this feature is not quite precise. It is hard to compute the number of basic sites, because it is complicated by the following factors: (1) it is possible that the mass differences between many pairs of peaks correspond to one same basic site, because 6 kinds of ions can be generated in CID although they are not equally likely generated. Besides, those ions can produce variants by losing water, ammonia, CO or NH group. (2) When we compute the number of basic sites, we don’t want to consider too much about their positions in a sequence, otherwise, it would become another complex problem, peptide de novo sequencing. However, when there are multiple basic sites especially multiple same basic sites like two K or two R existing in a peptide, we need to find a way to differentiate these two K or two R. (3) Situations when tryptic peptides end with two adjacent basic sites (KK, RR, KR, RK, HK, HR) or start with a basic site also complicate the computation. The research in [14] shows that when two basic sites are adjacent, it is more possible that only one of them can attach protons because there exists strong Coulombic repulsion force between adjacent protons. In addition, peptides start with basic residues will make the N-terminal amine group attract protons less likely, because the side chains of basic residues have much higher proton affinities than the amine group [14].

According to the definition of *n*_bs_, we can approach its computation in two possible ways: (1) compute the pseudo-number of basic sites by counting the number of all cases corresponding to a basic site and ignoring duplicate cases. This is reasonable because the pseudo-number of triply charged peptides should be generally larger than that of doubly charged ones. (2) figure out the theoretical repeat number of basic sites with the statistics of mass spectrometry generating ions. There is some research conducted to quantify the percentage of each kind of ion produced in CID. The study [15] reports some of such statistics based on the yeast proteome. However, data in a more general sense is needed. With the statistics of ions produced in CID, we can compute a theoretical repeat number for each basic residue. Then, it can be combined with the pseudo-number to derive the real number of basic sites in a mass spectrum. In this study, the feature *n*_bs_ was computed as the pseudo-number and transformed to have the variance 1. This feature is cogent in theory to discriminate doubly and triply charged MS/MS, but how to precisely compute it is still an open problem.

## Conclusions

A new approach for assigning charge states to low-resolution CID MS/MS is proposed based on the unsupervised GMM with four novel and discriminant features extracted from MS/MS. ROC and AUC demonstrate that GMM with proposed features is very promising in classifying doubly and triply charged MS/MS. For the future work, we will examine more on the computation of the number of basic sites, which theoretically should be the most significant feature in discriminating peptides with different charges.

## Authors' contributions

JS developed the algorithm, designed and executed all experimental work, and wrote the first draft. FXW supervised and initiated the project, and revised the manuscript. Both authors read and approved the manuscript.

## Competing interests

The authors declare that they have no competing interests.
